# Long-term immunosuppressive treatment is not associated with worse outcome in patients hospitalized in the intensive care unit for septic shock: the PACIFIC study

**DOI:** 10.1186/s13054-023-04626-z

**Published:** 2023-09-02

**Authors:** Julien Vaidie, Edwige Peju, Louise-Marie Jandeaux, Mathieu Lesouhaitier, Jean-Claude Lacherade, Antoine Guillon, Xavier Wittebole, Pierre Asfar, Bruno Evrard, Thomas Daix, Philippe Vignon, Bruno François

**Affiliations:** 1 Réanimation Polyvalente, CHU de Limoges, 2 Avenue Martin Luther King, 87042 Limoges Cedex, France; 2 Service de Médecine Intensive et Réanimation, Hôpital Cochin, Assistance Publique-Hôpitaux de Paris, Paris, France; 3 Médecine Intensive et Réanimation, Nouvel Hôpital Civil, CHRU de Strasbourg, Strasbourg, France; 4 Service de Maladies Infectieuses et Réanimation Médicale, CHU de Rennes, Rennes, France; 5 Médecine Intensive – Réanimation, CHD Vendée, La Roche-sur-Yon, France; 6 Médecine Intensive – Réanimation, CHRU Bretonneau, Tours, France; 7 Service de Soins Intensifs, Cliniques universitaires Saint Luc, Brussels, Belgium; 8 Médecine Intensive – Réanimation et médecine hyperbare, CHU Angers, Angers, France; 9 Inserm CIC 1435, CHU Dupuytren, Limoges, France; 10 Inserm UMR 1092, CHU Dupuytren, Limoges, France; 11 Inserm UMR 1100, UFR de Médecine, Tours, France

**Keywords:** Septic shock, Intensive care unit, Immunosuppression therapy, Organ transplantation, Autoimmune diseases, Mortality

## Abstract

**Background:**

Except in a few retrospective studies mainly including patients under chemotherapy, information regarding the impact of immunosuppressive therapy on the prognosis of patients admitted to the intensive care unit (ICU) for septic shock is scarce. Accordingly, the PACIFIC study aimed to asses if immunosuppressive therapy is associated with an increased mortality in patients admitted to the ICU for septic shock.

**Methods:**

This was a retrospective epidemiological multicentre study. Eight high enroller centres in septic shock randomised controlled trials (RCTs) participated in the study. Patients in the “exposed” group were selected from the screen failure logs of seven recent RCTs and excluded because of immunosuppressive treatment. The “non-exposed” patients were those included in the placebo arm of the same RCTs. A multivariate logistic regression model was used to estimate the risk of death.

**Results:**

Among the 433 patients enrolled, 103 were included in the “exposed” group and 330 in the “non-exposed” group. Reason for immunosuppressive therapy included organ transplantation (n = 45 [44%]) or systemic disease (n = 58 [56%]). ICU mortality rate was 24% in the “exposed” group and 25% in the “non-exposed” group (*p* = 0.9). Neither in univariate nor in multivariate analysis immunosuppressive therapy was associated with a higher ICU mortality (OR: 0.95; [95% CI 0.56–1.58]: *p* =  0.86 and 1.13 [95% CI 0.61–2.05]: *p* =  0.69, respectively) or 3-month mortality (OR: 1.13; [95% CI 0.69–1.82]: *p* =  0.62 and OR: 1.36 [95% CI 0.78–2.37]: *p* =  0.28, respectively).

**Conclusions:**

In this study, long-term immunosuppressive therapy excluding chemotherapy was not associated with significantly higher or lower ICU and 3-month mortality in patients admitted to the ICU for septic shock.

**Supplementary Information:**

The online version contains supplementary material available at 10.1186/s13054-023-04626-z.

## Background

Despite better knowledge of the pathophysiology and a more homogenous and standardized management [1, 2], the mortality of patients with septic shock admitted in the intensive care unit (ICU) remains high around 35% [3]. Immunosuppression is increasingly present in severely ill patients hospitalized in the ICU for sepsis or septic shock and large epidemiological studies have estimated a prevalence between 20 and 25% [4–6]. Immunosuppressed population is highly heterogeneous due to different levels of immunosuppression depending on the underlying pathology and therapeutic class used. In patients without HIV or primary immunodeficiency, pre-admission immunosuppression is mostly related to chemotherapy for haematological malignancies or a solid tumour, and long-term immunosuppressive treatment for an organ transplantation or an autoimmune disease (e.g., long-term steroid therapy, calcineurin, mTOR, TNF inhibitors, or immunosuppressive monoclonal antibodies).

Immunosuppression might modulate the host response to infection and may participate in higher mortality [7], reaching up to 70% six months after ICU admission for septic shock especially in patients with cancer [8–10]. Large retrospective studies showed an association between systemic disease and decreased 30-day mortality among ICU patients with septic shock [11], or a decreased in-hospital mortality in solid organ transplant patients hospitalized for sepsis [12, 13]. Apart from these exceptions, information regarding the impact of long-term immunosuppressive treatment on the prognosis of patients admitted to the ICU for septic shock is scarce and only a few retrospective studies have been published, mainly including patients under chemotherapy [7, 14, 15], especially because this specific population is usually excluded from most clinical studies and therapeutic trials on sepsis.

Accordingly, the PACIFIC study aimed to assess if long-term immunosuppressive therapy, excluding chemotherapy, was associated with a significantly increased mortality in patients admitted to the ICU for septic shock.

## Methods

This retrospective epidemiological multicentre study involved 8 centres in France and Belgium. They all participated in randomized controlled trials (RCTs) assessing new drugs to treat septic shock, according to the Sepsis-3 definition [16], between 2015 and 2022 [ADRENOSS (NCT03085758), Sepsis-Act (NCT02508649), ART-123 (NCT01598831), REVIVAL (NCT04411472), IRIS7a (NCT02797431), MOT-C 201 (NCT03158948), MOT-C 203 (NCT04055909)]. These sites were selected as high enrollers in the above-mentioned trials, which all excluded immunocompromised patients. We used an original patient selection approach. Patients who were screened but excluded due to a long-term immunosuppressive therapy constituted our “exposed” group, and were identified from the screening failure logs of each trial. Immunosuppressive therapy included one or more of the following treatments for at least one month before the septic shock: steroids (at least 10 mg/day of prednisone or equivalent), calcineurin inhibitors, mTOR inhibitors, unspecific immunosuppressors, TNF inhibitors or immunosuppressive monoclonal antibodies (e.g., rituximab or infliximab). Patients with HIV or who had received chemotherapy within 6 months before ICU admission, an hematopoietic stem cell transplantation, or alemtuzumab (anti-CD52 mAb) were not included in our study, because of the depth of immunosuppression related to these drugs and the known poorer prognosis of these immunocompromised patients.

Patients allocated to the placebo arm (i.e., standards of care according to Surviving Sepsis Campaign) in one of the selected sepsis trials constituted the “non-exposed” group.

The ethics committee of the Limoges university hospital approved the study (N°532-2022-188) and waived the need for written informed consent. A written information was sent to patients included in the “exposed” group. Patients from the “non-exposed” group already agreed to the use of their personal data in their initial informed consent from the selected RCTs.

Data has been collected directly from patients’ medical charts in each centre. The following parameters were recorded: demographic characteristics (age, gender, comorbidities), infection site, organ support, Sequential Organ Failure Assessment (SOFA) score on ICU admission and Simplified Acute Physiology Score (SAPS) II at the time of RCT screening, ICU and 3–month all-cause mortality.

To increase the study power, we used a ratio of three "non-exposed" patients for one "exposed" with the aim to obtain 100 patients in the “exposed” group. In each group, patients were included consecutively and chronologically until predetermined group size was reached.

Qualitative variables are expressed as numbers and percentages and were compared using the Chi^2^ test. Quantitative variables are expressed as median and their interquartile range (IQR) and were compared using the Mann–Whitney test. A logistic regression model was used to estimate the risk of ICU death. The parameters known to be independent mortality factors were forced into the multivariate analysis (age, SOFA score on admission, SAPS II at the time of screening for participation in RCTs). Results are expressed as odds ratio (OR) with the 95% confidence intervals (CI).

## Results

**Fig. 1:**
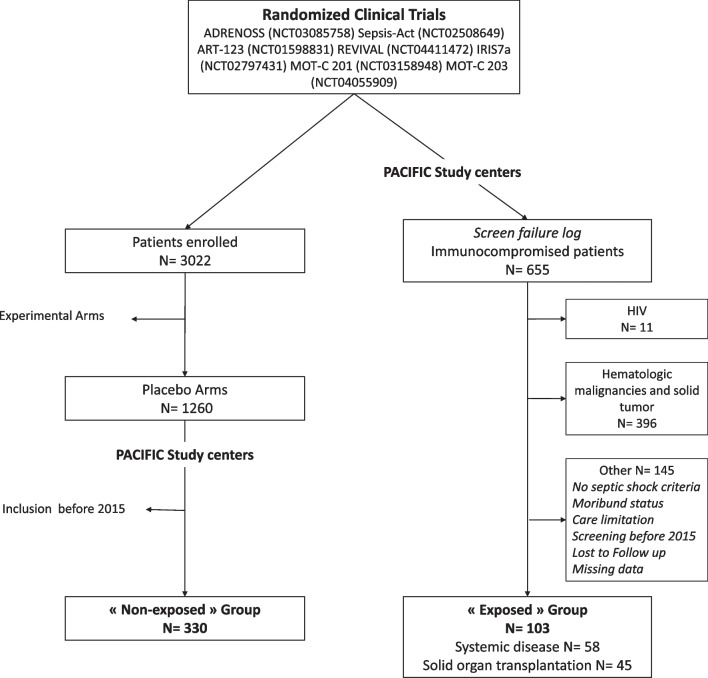
Flow chart

Four hundred and thirty-three patients were enrolled in the PACIFIC study. In the seven selected RCTs, 3022 patients were previously enrolled. Among them, 330 patients were selected to compose the “non-exposed” group with patients initially included in the placebo arms. One hundred and three patients were selected from the screen failure log to compose the “exposed” group (Fig. 1). In the "exposed" group, reason for immunosuppressive treatment was organ transplantation (n = 45 [44%]) or systemic disease (n = 58 [56%]). Patients with long-term immunosuppressive therapy were mainly men, had a lower SOFA score on ICU admission and the incidence of chronic renal failure was higher compared to the "non-exposed" patients (Table 1 and Additional file 1: Table S1).

**Table 1:** Patients characteristics

Characteristics	Total	Non-exposed (no immunosuppressive treatment)	Exposed (immunosuppressive treatment)	*p* value
	n = 433	n = 330	n = 103	
Age (years)	68 (59–76)	69 (60–77)	67 (58–76)	0.2
Men	277 (64%)	222 (67%)	55 (53%)	0.01
SOFA score at admission	10 (8–12)	10 (8–12)	9 (7–11)	0.01
SAPS II at screening	59 (47–71)	60 (47–71)	56 (48–66)	0.14
Comorbidities				
High blood pressure	230 (53%)	172 (52%)	58 (56%)	0.5
Diabetes	126 (29%)	95 (29%)	31 (30%)	0.8
Chronic heart failure	66 (15%)	50 (15%)	16 (16%)	> 0.9
Chronic respiratory failure	52 (12%)	40 (12%)	12 (12%)	0.9
Chronic renal failure	89 (21%)	48 (15%)	41 (40%)	< 0.001
Infection site				
Lungs	157 (36%)	118 (36%)	39 (38%)	
Abdomen	106 (24%)	85 (26%)	21 (20%)	
Urine	90 (21%)	66 (20%)	24 (23%)	
Skin	26 (6%)	22 (6.7%)	4 (3.9%)	
Central nervous system	9 (2.1%)	8 (2.4%)	1 (1%)	
Joints and bones	12 (2.8%)	6 (1.8%)	6 (5.8%)	
Bacteraemia	33 (7.6%)	25 (7.6%)	8 (7.8%)	
Cause of immunosuppression				
Organ transplantation	–	–	45 (44%)	
Systemic disease	–	–	58 (56%)	
Organ support and outcome				
ICU stay duration (days)	9 (5–15)	9 (5–15)	8 (4–14)	0.2
Number of days with vasopressor support	4 (2–9)	4 (2–10)	3 (1–7)	0.11
Number of mechanically ventilated patients	329 (76%)	259 (78%)	70 (68%)	0.029
Number of days with mechanical ventilation	4 (2–6)	4 (2–6)	4 (2–6)	0.7
Patients with dialysis during hospital stay	133 (31%)	90 (27%)	43 (42%)	0.005
ICU death	108 (25%)	83 (25%)	25 (24%)	0.9
Three-month mortality	126 (29%)	94 (28%)	32 (31%)	0.6

*SOFA* Sequential Organ Failure Assessment, *ICU* Intensive care unit

**Fig. 2:**
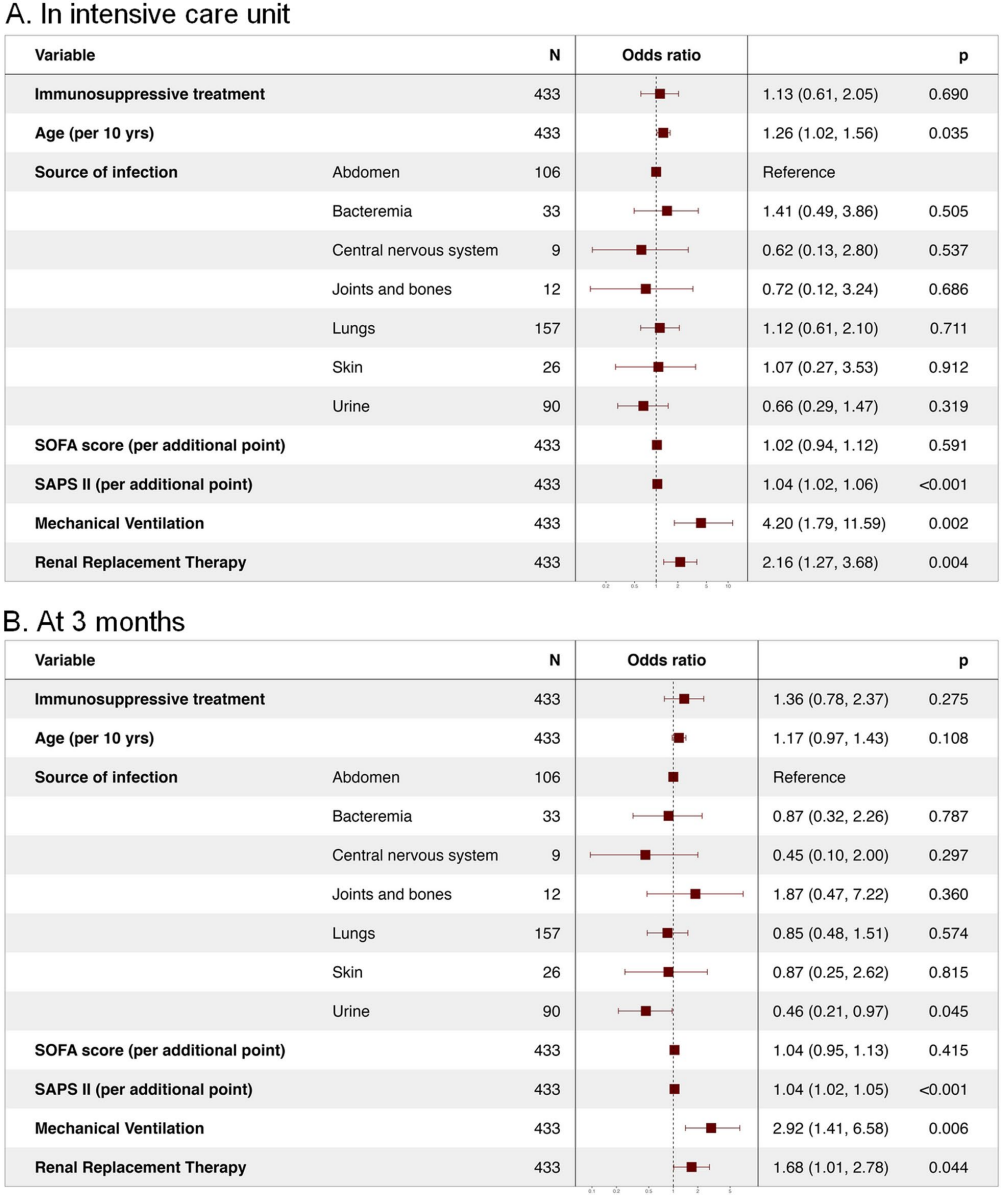
Multivariate analysis. SOFA = Sequential Organ Failure Assessment; SAPS II = Simplified Acute Physiology Score II

In the univariate analysis, long-term immunosuppressive treatment was not associated with a higher risk of ICU death (OR: 0.95; 95% CI 0.56–1.58; *p* = 0.86). In contrast, age (OR: 1.29 per 10 years; 95% CI 1.09–1.54; *p* = 0.003), SOFA score (OR: 1.14 per additional point; 95% CI 1.06–1.23; *p* < 0.001) and SAPS II score at the time of RCT screening (OR: 1.05 per additional point; 95% CI 1.04–1.07; *p* < 0.001) were significantly associated with an increased risk of ICU mortality. Invasive mechanical ventilation and dialysis were also associated with an increased risk of death (Additional file 1: Table S2). In the multivariate analysis, long-term immunosuppressive treatment was not associated with an increased risk of ICU mortality (OR: 1.13; 95% CI 0.61–2.05; *p* = 0.69). In contrast, SAPS II and invasive mechanical ventilation or dialysis remained associated with a higher risk of ICU mortality (Fig. 2).

In the multivariate analysis, long-term immunosuppressive treatment was not associated with increased three-month mortality, with an OR at 1.36 (95% CI 0.78–2.37; *p* = 0.28). In contrast, SAPS II remained associated with an increased three-month mortality risk (Fig. 2).

In the multivariate subgroup analysis, the underlying immunosuppression, namely systemic disease or solid organ transplant, was not associated with ICU (OR at 1.81 [95% CI 0.85–3.77] and 0.59 [95% CI 0.22–1.43] respectively; *p* = 0.13) and three-month mortality (OR at 1.74 [95% CI 0.86–3.48] and 0.98 [95% CI 0.96–1.41] respectively; *p* = 0.29) (Additional file 1: Table S2 and Figs. S1 and S2).

ICU length of stay was similar in the “exposed” and “non-exposed” groups (8 ± 4 vs. 9 ± 5 days: *p* = 0.2) as was the number of vasopressor-free days within 30 days (27 ± 2 days vs. 26 ± 3 days: *p* = 0.11). In contrast, a higher proportion of patients required mechanical ventilation in the "non-exposed" group compared to the "exposed" group (259/330 [78%] vs. 70/103 [68%]; *p* = 0.029), but the duration of ventilation was similar between groups (4 ± 2 vs. 4 ± 2 days: *p* = 0.7). A significantly higher proportion of "exposed" patients required dialysis (90/330 [27%] vs. 43/103 [42%]; *p* = 0.005) (Table 1).

## Discussion

The present study mainly shows that patients receiving long-term immunosuppressive drugs who were hospitalized in the ICU for septic shock did not have a higher mortality when compared to non-immunosuppressed patients. Not surprisingly, age and severity score on ICU admission were independently associated with ICU and three-month mortality.

To our knowledge, the PACIFIC study is the first that specifically targeted patients with septic shock who were exposed to long-term immunosuppressive treatment and hospitalized in ICU. To better identify the potential effect of immunosuppressive treatment on outcome, we used an original selection approach to reduce biases inherent to classical study design such as retrospective cohort or case control studies. Namely, we selected patients with homogenously defined septic shock (according to Sepsis-3 definition) who fulfilled the same eligibility criteria to participate in RCTs assessing the effects of newly developed agents during a given time period. To limit selection bias, patients were consecutively and chronologically enrolled in the present study, whether they had been enrolled in the placebo arm of selected RCTs or excluded due to long-term immunosuppressive treatment. To follow the same approach as in studies in patients with cancer (regardless of the type, stage or therapeutic class), we highlighted the effect of the immunosuppressive treatment by including a heterogeneous population, thus limiting the effect of each underlying condition and each therapeutic class.

In the “non-exposed” group, ICU and three-month mortality only reached 25% and 28% respectively, while a recent meta-analysis reported, between 2009 and 2019, a one-month and three-month mortality at 34.7% et 38.5% respectively in septic shock [3]. The relatively lower mortality observed in the present study may be related to the enrolment approach, i.e. from recent RCTs conducted in septic shock. It might have created a selection bias since the most severe patients (i.e., life expectancy shorter than 48h and moribund status) are most of the time excluded from these trials. In the “exposed” group, ICU mortality reached 24%. No reliable estimation corresponding to our specific study population has been published yet. Publications related to immunosuppressed patients mostly included patients receiving chemotherapy for malignancies, with sepsis or septic shock, without taking into account specifically the Sepsis-3 definition. Nevertheless, subgroup analyses from these studies reported a mortality rate between 31 and 51% in immunocompromised patients without chemotherapy [7, 14, 15]. Even though the mortality rate is similar between the two groups, it does not reflect the percentage and absolute mortality in the general population, in particular in the “exposed” group known to be more susceptible to infection and sepsis.

Although the study population and inclusion criteria differed from the PACIFIC study as previously mentioned, our results are in contrast with other studies which showed a decreased mortality rate in patients under long-term immunosuppressive treatment hospitalized for sepsis. Sheth et al. [11] showed a statistically significant decrease of 30-day mortality in patients with autoimmune diseases under long-term immunosuppressive treatment who were hospitalized for sepsis or septic shock, when compared to the general population (OR: 0.73; 95% CI 0.57–0.93). Colbert et al. [17] studied patients with inflammatory bowel disease and reported a decreased risk of hospital mortality in patients with Crohn's disease admitted for sepsis or septic shock (OR: 0.78; 95% CI 0.63–0.97). In solid organ transplant recipients hospitalized for sepsis, Donelly et al. [12] reported a decreased in-hospital mortality (OR: 0.83; 95% CI 0.79–0.87) in a large retrospective study, and so did Kalil et al. [18] at day 28 and day 90 in sepsis with bacteremia (HR: 0.22; 95% CI 0 0.09–0.54 and 0.43; 95% CI 0.20–0.89, respectively) or, more recently, Ackermann et al. [13]. Finally, in line with our results, the single-centre retrospective study by Jamme et al. [7], conducted in 309 immunocompromised patients admitted in the ICU for septic shock, reported an increased risk of hospital mortality in patients with solid tumour, but not in the subgroup of patients without malignancies (OR: 1.35; 95% CI 0.92–1.98), when compared to the general population. These differences might be explained by a lack of power despite our substantial sample size. On another hand, the new definition of septic shock that we used may have detected a specific patient profile different from the population of the previously mentioned studies.

Host dysregulated immune response, which characterises sepsis, combines pro- and anti-inflammatory mechanisms [19]. The absence of significant impact of long-term immunosuppressive therapy on ICU and three-month mortality in our patients may appear counter-intuitive and not in agreement with previously published studies conducted in cancer patients [8, 10]. The entire immune system is affected during sepsis, with pro-apoptotic effects of B and T lymphocytes, and alteration of myeloid cells, especially monocytes, due to a decrease in HLA-DR expression [19, 20]. There is currently little data available regarding the impact of sepsis on the immune system of immunocompromised patients. Many studies have suggested a link between the prognosis of patients and different cytokine profiles observed during the acute phase of septic shock [21, 22]. In autoimmune diseases, Sheth et al. [11] hypothesized that cytokine profiles were different depending on the type of the underlying autoimmune disease and that some profiles could potentially protect from septic shock-related mortality, especially in patients with high expression of IL-12 or IFN-γ. It can also be assumed that exposure to immunosuppressive treatments protects from the excessive and potentially deleterious inflammatory response that characterises septic shock [16]. The class of immunosuppressive treatment used may also alter the modulation of this cytokine response differently. In addition, long-term steroid therapy and substitution may counteract the effect against the development of the relative adrenocortical insufficiency documented in some patients [23–25]. Managing immunosuppressive treatments is a major issue in septic shock, and how to enhance immunity response limiting organ rejection or systemic disease exacerbation [26]. Then, the sepsis-induced immunosuppression phase that has been described in patients not previously exposed to an immunosuppressive treatment, may result in an immune profile similar to that of the exposed patients [19, 27, 28]. The aggression induced by septic shock in immunocompetent patients could thus lead to a dysregulation of the immune system that is similar to that of patients under long-term immunosuppressive therapy, thus providing a similar risk of mortality [28].

Our study has several limitations. Despite a study design to limit selection bias, this approach generated its own groups’ characteristics with several baseline differences and management (i.e. men ratio, SOFA at admission, chronic renal insufficiency rate, dialysis during hospitalization, mechanical ventilation rate) which could interfere with the two groups comparability and the external validity. Then, in the multivariate analysis, age and SOFA score were not associated with increased mortality in sepsis as they are usually. Concerning septic shock management, we did not collect comprehensive information about antibiotic therapy administration (i.e. time between hospital admission and drug delivery, appropriateness of the first-line antibiotics, duration of antibiotic therapy…), which could constitute a bias for the interpretation of results. In addition, there is no consensus to reduce administration or temporary withdraw immunosuppressive agents, but different management strategies might be another source of bias. Most importantly, the magnitude of confidence intervals in the multivariate analysis allows for uncertainty, so that long-term immunosuppressive treatment could be associated with either benefit or harm. Finally, the heterogeneity of the “exposed” group encourages careful interpretation of the results, since the underlying condition and therapeutic class used for each patient could be different with possibly different consequences on the outcome.

Including patients with immunosuppressive treatments in RCTs assessing new drugs in septic shock, especially when following the inflammatory and cytokine profiles of patients over time, would greatly contribute to expand the currently scarce clinical data on this growing population and improve the existing gap of knowledge in pathophysiology.

## Conclusion

This multicentre retrospective study did not evidence a higher or lower mortality in patients exposed to a long-term immunosuppressive treatment when compared to the non-immunosuppressed population admitted to the ICU for septic shock. Accordingly, excluding immunocompromised patients from RCTs assessing new drugs in sepsis or septic shock could be cautiously reconsidered.

### Supplementary Information

**Additional file 1: Table S1.** Patients’ characteristics – supplementary data. **Table S2.** Univariate and Multivariate Analysis. **Figure S1.** Intensive care unit Subgroup Multivariate Analysis. **Figures S2.** 3-month Subgroup Multivariate Analysis.

## Data Availability

The datasets used and/or analysed during the current study are available from the corresponding author on reasonable request.

## Abbreviations

CI: Confidence intervals
HIV: Human immunodeficiency virus
ICU: Intensive care unit
IQR: Interquartile range
OR: Odds ratio
RCT: Randomised controlled trial
SAPS II: Simplified Acute Physiology Score II
SOFA: Sequential Organ Failure Assessment

## References

[1] Rhodes A, Evans LE, Alhazzani W, Levy MM, Antonelli M, Ferrer R (2017). Surviving sepsis campaign: international guidelines for management of sepsis and septic shock: 2016. Intensive Care Med.

[2] Evans L, Rhodes A, Alhazzani W, Antonelli M, Coopersmith CM, French C (2021). Surviving sepsis campaign: international guidelines for management of sepsis and septic shock 2021. Intensive Care Med.

[3] Bauer M, Gerlach H, Vogelmann T, Preissing F, Stiefel J, Adam D (2020). Mortality in sepsis and septic shock in Europe, North America and Australia between 2009 and 2019-results from a systematic review and meta-analysis. Crit Care Lond Engl.

[4] Annane D, Aegerter P, Jars-Guincestre MC, Guidet B, CUB-Réa Network. Current epidemiology of septic shock: the CUB-Réa Network. Am J Respir Crit Care Med. 2003;168:165–72. 10.1164/rccm.2201087 12851245

[5] Vincent J-L, Sakr Y, Sprung CL, Ranieri VM, Reinhart K, Gerlach H (2006). Sepsis in European intensive care units: Results of the SOAP study*. Crit Care Med.

[6] Vincent J-L, Sakr Y, Singer M, Martin-Loeches I, Machado FR, Marshall JC (2020). Prevalence and outcomes of infection among patients in intensive care units in 2017. JAMA.

[7] Jamme M, Daviaud F, Charpentier J, Marin N, Thy M, Hourmant Y (2017). Time course of septic shock in immunocompromised and nonimmunocompromised patients. Crit Care Med.

[8] Rosolem MM, Rabello LSCF, Lisboa T, Caruso P, Costa RT, Leal JVR (2012). Critically ill patients with cancer and sepsis: clinical course and prognostic factors. J Crit Care.

[9] Azoulay E, Schellongowski P, Darmon M, Bauer PR, Benoit D, Depuydt P (2017). The intensive care medicine research agenda on critically ill oncology and hematology patients. Intensive Care Med.

[10] Azoulay E, Mokart D, Pène F, Lambert J, Kouatchet A, Mayaux J (2013). Outcomes of critically Ill patients with hematologic malignancies: prospective multicenter data from France and Belgium—a groupe de recherche respiratoire en réanimation onco-hématologique study. J Clin Oncol.

[11] Sheth M, Benedum CM, Celi LA, Mark RG, Markuzon N (2019). The association between autoimmune disease and 30-day mortality among sepsis ICU patients: a cohort study. Crit Care Lond Engl.

[12] Donnelly JP, Locke JE, MacLennan PA, McGwin G, Mannon RB, Safford MM (2016). Inpatient mortality among solid organ transplant recipients hospitalized for sepsis and severe sepsis. Clin Infect Dis Off Publ Infect Dis Soc Am.

[13] Ackerman KS, Hoffman KL, Díaz I, Simmons W, Ballman KV, Kodiyanplakkal RP (2023). Effect of sepsis on death as modified by solid organ transplantation. Open Forum Infect Dis.

[14] Poutsiaka DD, Davidson LE, Kahn KL, Bates DW, Snydman DR, Hibberd PL (2009). Risk factors for death after sepsis in patients immunosuppressed before the onset of sepsis. Scand J Infect Dis.

[15] Tolsma V, Schwebel C, Azoulay E, Darmon M, Souweine B, Vesin A (2014). Sepsis severe or septic shock: outcome according to immune status and immunodeficiency profile. Chest.

[16] Singer M, Deutschman CS, Seymour CW, Shankar-Hari M, Annane D, Bauer M (2016). The third international consensus definitions for sepsis and septic shock (sepsis-3). JAMA.

[17] Colbert JF, Schmidt EP, Faubel S, Ginde AA (2017). Severe sepsis outcomes among hospitalizations with inflammatory bowel disease. Shock Augusta Ga.

[18] Kalil AC, Syed A, Rupp ME, Chambers H, Vargas L, Maskin A (2015). Is bacteremic sepsis associated with higher mortality in transplant recipients than in nontransplant patients? A matched case-control propensity-adjusted study. Clin Infect Dis Off Publ Infect Dis Soc Am.

[19] Hotchkiss RS, Monneret G, Payen D (2013). Sepsis-induced immunosuppression: from cellular dysfunctions to immunotherapy. Nat Rev Immunol.

[20] Gustave C-A, Gossez M, Demaret J, Rimmelé T, Lepape A, Malcus C (1950). Septic shock shapes B cell response toward an exhausted-like/immunoregulatory profile in patients. J Immunol Baltim Md.

[21] Scicluna BP, van Vught LA, Zwinderman AH, Wiewel MA, Davenport EE, Burnham KL (2017). Classification of patients with sepsis according to blood genomic endotype: a prospective cohort study. Lancet Respir Med.

[22] Tsalik EL, Langley RJ, Dinwiddie DL, Miller NA, Yoo B, van Velkinburgh JC (2014). An integrated transcriptome and expressed variant analysis of sepsis survival and death. Genome Med.

[23] Annane D, Sébille V, Charpentier C, Bollaert P-E, François B, Korach J-M (2002). Effect of treatment with low doses of hydrocortisone and fludrocortisone on mortality in patients with septic shock. JAMA.

[24] Annane D, Renault A, Brun-Buisson C, Megarbane B, Quenot J-P, Siami S (2018). Hydrocortisone plus fludrocortisone for adults with septic shock. N Engl J Med.

[25] Pourmand A, Whiteside T, Yamane D, Rashed A, Mazer-Amirshahi M (2019). The controversial role of corticosteroids in septic shock. Am J Emerg Med.

[26] Bafi AT, Tomotani DYV, de Freitas FGR (2017). Sepsis in solid-organ transplant patients. Shock Augusta Ga.

[27] Venet F, Monneret G (2018). Advances in the understanding and treatment of sepsis-induced immunosuppression. Nat Rev Nephrol.

[28] van Vught LA, Klein Klouwenberg PMC, Spitoni C, Scicluna BP, Wiewel MA, Horn J (2016). Incidence, risk factors, and attributable mortality of secondary infections in the intensive care unit after admission for sepsis. JAMA.

