# Supplementary material for: Long-term immunosuppressive treatment is not associated with worse outcome in patients hospitalized in the intensive care unit for septic shock: the PACIFIC study
Source: Crit Care. 2023 Sep 2;27:340. doi: 10.1186/s13054-023-04626-z (PMC10475175; doi:10.1186/s13054-023-04626-z)

Supplementary appendix

Supplementary Table 1: Patients' characteristics – supplementary data

Characteristics	Total<br>n = 433	Non-exposed (no immunosuppressive treatment)<br>n = 330	Exposed (immunosuppressive treatment)<br>n = 103	p
**Blood count at inclusion**				
Leukocytes (Giga/L)	13 (7-22)	14 (7-22)	12 (6-20)	0.1
Lymphocytes (Giga/L)	0.62 (0.35-1.03)	0.65 (0.37-1.08)	0.56 (0.29-0.90)	0.12
Platelets (Giga/L)	172 (118-247)	176 (119-262)	160 (112-214)	0.066
**Cause of immunosuppression**				
Organ transplantation	-	-	45 (44%)	
Systemic disease	-	-	58 (56%)	
Connective			15 (14%)	
Rheumatoid arthritis			26 (25%)	
Inflammatory bowel disease			6 (6%)	
Vasculitis			3 (3%)	
Sarcoidosis			3 (3%)	
Other			5 (5%)	
**Immunosuppressive drugs**				
Corticosteroids	-	-	91 (88%)	
Calcineurin inhibitors	-	-	35 (34%)	
mTOR inhibitors			10 (10%)	
Mycophenolate mofetil	-	-	45 (44%)	
Rituximab	-	-	5 (5%)	
TNF inhibitors	-	-	6 (6%)	
Methotrexate	-	-	8 (8%)	
Cyclophosphamide	-	-	4 (4%)	
Azathioprine	-	-	4 (4%)	

Supplementary Table 2 – Univariate and Multivariate Analysis

			Univariate analysis			Multivariate analysis			Multivariate analysis (Subgroup)		
**Characteristics**	**n**	**Death**	**OR**	**95% CI**	**p**	**OR**	**95% CI**	**p**	**OR**	**95% CI**	**p**
**In intensive care unit**											
**Age**	433	108	1.29*	1.09-1.54	0.003	1.26*	1.02-1.57	0.03	1.23*	1.00-1.53	0.055
**Sex (Male)**	433	108	1.47	0.92-2.37	0.11						
**High blood pressure**	433	108	1.08	0.70-1.68	0.72						
**Diabetes**	433	108	1.31	0.81-2.08	0.27						
**Chronic Respiratory Failure**	433	108	1.71	0.91-3.14	0.10						
**Chronic renal failure**	433	108	0.84	0.48-1.44	0.54						
**Chronic Heart Failure**	433	108	1.63	0.92-2.85	0.10						
**SOFA score at admission**	433	108	1.14**	1.06-1.23	<0.001	1.02**	0.94-1.12	0.59	1.03**	0.95-1.13	0.46
**SAPS II**	433	108	1.05**	1.04-1.07	<0.001	1.04**	1.02-1.06	<0.001	1.04**	1.02-1.06	<0.001
**Infection site**	433	108			0.24			0.79			0.73
Abdominal				Reference			Reference			Reference	
Bacteraemia			1.04	0.42-2.46		1.41	0.49-3.86		1.27	0.44-3.51	
CNS			2.23	0.52-9.01		0.62	0.13-2.80		0.55	0.11-2.53	
Skin			0.51	0.14-1.47		1.07	0.27-3.53		1.13	0.29-3.77	
Joints and bones			0.93	0.20-3.37		0.72	0.12-3.24		0.55	0.09-2.57	
Lungs			1.12	0.65-1.96		1.12	0.61-2.10		1.11	0.60-2.09	
Urine			0.56	0.27-1.11		0.66	0.29-1.47		0.65	0.28-1.43	
**Mechanical Ventilation**	433	108	7.34	3.37-19.3	<0.001	4.20	1.79-11.6	<0.001	4.37	1.84-12.2	<0.001
**Dialysis**	433	108	3.11	1.98-4.92	<0.001	2.16	1.27-3.68	0.004	2.41	1.40-4.17	0.002
**Immunosuppressive treatments**	433	108	0.95	0.56-1.58	0.86	1.13	0.61-2.05	0.69			
**Underlying Immunosuppression**	433	108			0.45						0.13
**Systemic disease**			1.20	0.64-2.20					1.81	0.85-3.77	
**Solid organ transplant**			0.66	0.28-1.41					0.59	0.22-1.43	
**At 3 months**											
**Age**	433	126	1.22*	1.04-1.44	0.012	1.17*	0.97-1.43	0.10	1.16*	0.96-1.41	0.14
**Sex (Male)**	433	126	1.24	0.80-1.94	0.33						
**High blood pressure**	433	126	0.96	0.63-1.45	0.84						
**Diabetes**	433	126	1.26	0.80-1.97	0.32						
**Chronic Respiratory Failure**	433	126	1.78	0.97-3.22	0.063						
**Chronic renal failure**	433	126	1.01	0.60-1.67	0.98						
**Chronic Heart Failure**	433	126	1.61	0.92-2.76	0.095						
**SOFA score at admission**	432	125	1.13**	1.05-1.22	<0.001	1.04**	0.95-1.13	0.42	1.04**	0.96-1.13	0.35
**SAPS II**	433	126	1.05**	1.03-1.06	<0.001	1.04**	1.02-1.05	<0.001	1.04**	1.02-1.05	<0.001
**Infection site**	433	126			0.051			0.35			0.36
Abdominal				Reference			Reference			Reference	
Bacteraemia			0.73	0.29-1.69		0.87	0.32-2.26		0.82	0.30-2.15	

CNS			1.56	0.37-6.23		0.45	0.10-2.00		0.42	0.09-1.90	
Skin			0.46	0.15-1.25		0.87	0.25-2.62		0.89	0.26-2.69	
Joints and bones			1.94	0.57-6.64		1.87	0.47-7.22		1.64	0.40-6.46	
Lungs			0.91	0.54-1.54		0.85	0.48-1.51		0.84	0.47-1.50	
Urine			0.42	0.21-0.81		0.46	0.21-0.97		0.45	0.21-0.95	
**Mechanical Ventilation**	433	126	5.12	2.68-10.8	<0.001	2.92	1.41-6.56	0.006	2.94	1.42-6.66	0.003
**Dialysis**	433	126	2.64	1.71-4.09	<0.001	1.71	1.03-2.83	0.039	1.77	1.06-2.97	0.030
**Immunosuppressive treatments**	433	126	1.13	0.69-1.82	0.62	1.36	0.78-2.37	0.28			
**Underlying Immunosuppression**	433	126			0.68						0.29
**Systemic disease**			1.29	0.70-2.30					1.74	0.86-3.48	
**Solid organ transplant**			0.94	0.45-1.86					0.98	0.42-2.17	

*: per additional ten years; **: per additional point.

SOFA = Sequential Organ Failure Assessment; SAPS II = Simplified Acute Physiology Score II

Supplementary Figure 1 – Intensive care unit Subgroup Multivariate Analysis

Variable		N	Odds ratio		p
**Immunosuppressive treatments**	No immunosuppression	330		Reference	
	Systemic disease	59		1.81 (0.85, 3.77)	0.118
	Solid organ transplant	44		0.59 (0.22, 1.43)	0.259
**Age (per 10 yrs)**		433		1.23 (1.00, 1.53)	0.062
**Source of infection**	Abdomen	106		Reference	
	Bacteremia	33		1.27 (0.44, 3.51)	0.649
	Central nervous system	9		0.55 (0.11, 2.53)	0.444
	Joints and bones	12		0.55 (0.09, 2.57)	0.474
	Lungs	157		1.11 (0.60, 2.09)	0.737
	Skin	26		1.13 (0.29, 3.77)	0.845
	Urine	90		0.65 (0.28, 1.43)	0.289
**SOFA score (per additional point)**		433		1.03 (0.95, 1.13)	0.457
**SAPS II (per additional point)**		433		1.04 (1.02, 1.06)	<0.001
**Mechanical Ventilation**		433		4.37 (1.84, 12.19)	0.002
**Renal Replacement Therapy**		433		2.41 (1.40, 4.17)	0.002

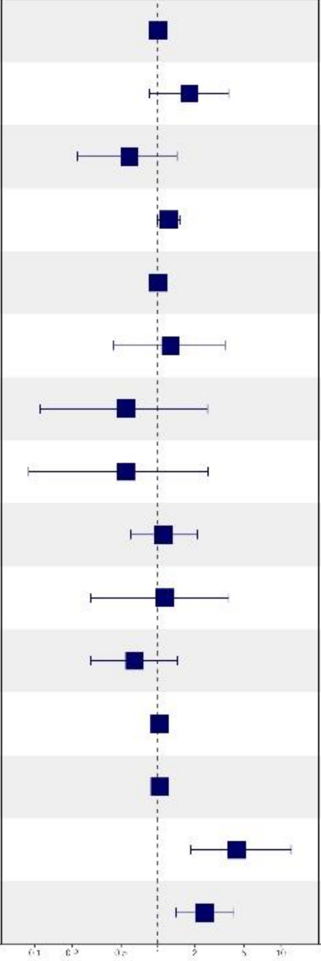

Supplementary Figure 2 – 3-month Subgroup Multivariate Analysis

Variable		N	Odds ratio	p
**Immunosuppressive status**	No immunosuppression	330	Reference	
	Systemic disease	59	1.74 (0.86, 3.48)	0.117
	Solid organ transplant	44	0.98 (0.42, 2.17)	0.962
**Age (per 10 yrs)**		433	1.16 (0.96, 1.41)	0.145
**Infection**	Abdomen	106	Reference	
	Bacteraemia	33	0.82 (0.30, 2.15)	0.699
	Central nervous system	9	0.42 (0.09, 1.90)	0.264
	Joints and bones	12	1.64 (0.40, 6.46)	0.479
	Lungs	157	0.84 (0.47, 1.50)	0.551
	Skin	26	0.89 (0.26, 2.69)	0.848
	Urine	90	0.45 (0.21, 0.95)	0.040
**SOFA score (per additional point)**		433	1.04 (0.96, 1.13)	0.354
**SAPS II (per additional point)**		433	1.04 (1.02, 1.05)	<0.001
**Mechanical Ventilation**		433	2.94 (1.42, 6.66)	0.006
**Renal Replacement Therapy**		433	1.77 (1.06, 2.97)	0.029

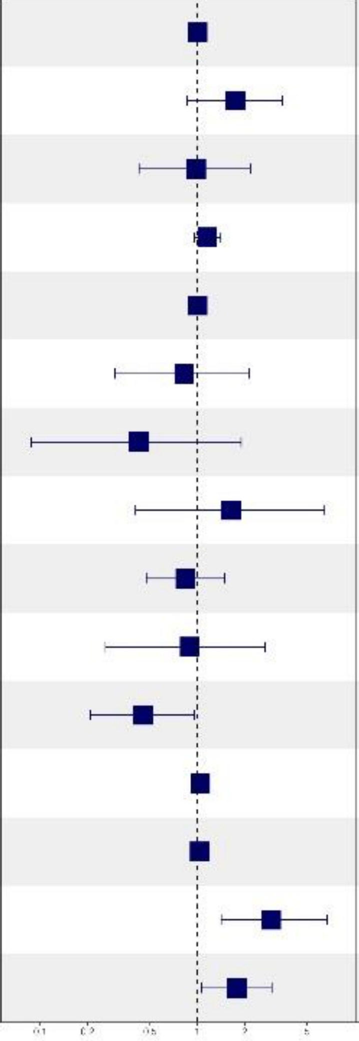

Supplement: Supplementary file 1 — Additional file 1: Table S1. Patients’ characteristics – supplementary data. Table S2. Univariate and Multivariate Analysis. Figure S1. Intensive care unit Subgroup Multivariate Analysis. Figures S2. 3-month Subgroup Multivariate Analysis. [file 13054_2023_4626_MOESM1_ESM.pdf]
